# Perceptions and experiences of community-based healthcare professionals in the state of Qatar having do not attempt resuscitation discussions during the COVID-19 pandemic

**DOI:** 10.3389/fmed.2023.1232954

**Published:** 2023-12-14

**Authors:** Audrey Fitzgerald, Conor Fitzgerald, Louise Anderson, Ammar Ali Hussain, Guillaume Alinier

**Affiliations:** ^1^Hamad Medical Corporation, Home Healthcare Services, Doha, Qatar; ^2^Hamad Medical Corporation Ambulance Service, Doha, Qatar; ^3^University of Hertfordshire, Hatfield, United Kingdom; ^4^Weill Cornell Medicine-Qatar, Doha, Qatar; ^5^Northumbria University, Newcastle upon Tyne, United Kingdom

**Keywords:** do not attempt resuscitation, COVID-19 pandemic, community medicine, Qatar, religion

## Abstract

**Introduction:**

The values and attitudes of healthcare professionals influence their handling of “do-not-attempt-resuscitation” (DNAR) orders, as does that of the families they interact with. The aim of this study was to describe attitudes, perceptions, and practices among community-based medical practitioners towards discussing cardiopulmonary resuscitation and DNAR orders with patients and their relatives, and to investigate if the COVID-19 pandemic affected their practice in having these discussions.

**Methods:**

This is a researcher-developed online survey-based study which aimed to recruit a convenience sample of respondents from a total population of 106 healthcare professionals working for the Mobile Healthcare Service (MHS), Hamad Medical Corporation Ambulance Service in the State of Qatar.

**Results:**

33 family physicians, 38 nurses, and 20 paramedics (*n* = 91) responded to the questionnaire, of who around 40, 8, and 50%, respectively, had engaged in Do Not Attempt Resuscitation discussions during their work with MHS. 15% of physicians who had experience with Do Not Attempt Resuscitation discussions in Qatar felt that the family or patient were not open to having such discussions. 90% of paramedics thought that Do Not Attempt Resuscitation was a taboo topic for their patients in Qatar, and this view was shared by 75% of physicians and 50% of nurses. Per the responses, the COVID-19 pandemic had not affected the likelihood of most of the physicians or nurses (and 50% of the paramedics) identifying patients with whom having a Do Not Attempt Resuscitation discussion would be clinically appropriate.

**Discussion:**

Overall, for all three groups, the COVID-19 pandemic did not affect the likelihood of identifying patients with whom a Do Not Attempt Resuscitation discussion would be clinically appropriate. We found that the greatest barriers in having Do Not Attempt Resuscitation discussions were perceived to be the religious or cultural beliefs of the patient and/or their family, along with the factor of feeling the staff member did not know the patient or their family well enough. All three groups said they would be more likely to have a conversation about Do Not Attempt Resuscitation if barriers were addressed.

## Introduction

A “Do-not-attempt-resuscitation” order, termed “DNAR or DNR”, is an order placed in a patient’s medical record to direct the healthcare team to withhold resuscitative measures. Do Not Attempt Resuscitation orders apply in any care setting, in or out of hospital, within the constraints of the applicable law (AMA Code of Medical Ethics).

The sensitive subject-matter of discussing and deciding on Do Not Attempt Resuscitation orders requires great skill, alongside medical and ethical judgment to balance the needs of the patient and their families. Alongside this, one needs to consider what is medically “doable”, what should be done or should not be done, either from a medical or ethical stand-point, and, of course, take into consideration what actions or decisions are permissible under the law.

In the Gulf Cooperation Council (GCC) states, these already difficult decisions are over-layered with religious and cultural beliefs that must also be at the forefront of the mind of the care pathway decision-makers (1). The laws that govern end of life stage care in the GCC are rooted in Islamic principles. Islam considers that life is sacrosanct and that efforts to treat a patient should be continued; traditionally treatment options would continue notwithstanding that the end result would be that the patient would eventually die. There has been a significant change in this approach in recent times.

Previous studies have indicated variable perceptions and experiences among physicians and nurses of the complex Do Not Attempt Resuscitation decision process (2–5). Confusion and difficulties regarding the Do Not Attempt Resuscitation decision can often arise despite the efforts of healthcare professionals (HCP) to help the patient and/or family to make an informed decision (6). Barriers to Do Not Attempt Resuscitation discussions include inconsistent documentation, the prevailing legislation of a jurisdiction, and conflicts regarding the best interests of the patient between the patient’s family members and the attending medical professionals (7–9).

The Islamic religion’s concept concerning Do Not Attempt Resuscitation decisions has been clarified by the Presidency of the Administration of Islamic Research and Ifta, Riyadh, KSA, in their Fatwa No. 12086 issued on 28/3/1409 (1989). The Fatwa states that: “if three knowledgeable and trustworthy physicians agreed that the patient condition is hopeless; the life-supporting machines can be withheld or withdrawn. The family members’ opinion is not included in decision-making as they are unqualified to make such decisions” (10, 11).

The right of terminally ill patients to give advanced directives related to their treatment such as that of a Do Not Attempt Resuscitation order has been instituted in Qatar’s Palliative Care Protocol since 2004. Following further revisions, the major public health institutions in Qatar, Hamad Medical Corporation (HMC) and the Primary Healthcare Corporation (PHCC) have now incorporated the protocol. HMC defines this policy as a legal right for a patient to choose, given the circumstance of deterioration or imminent collapse, to be withheld from the following interventions: chest compression, defibrillation, endotracheal intubation, assisted ventilation, and administration of cardiac drugs (12).

In Qatar, three physicians need to sign the Do Not Attempt Resuscitation order if they think it is clinically appropriate. Patients or their family members do not need to sign. However, hospital regulations allow it only after discussion with and agreement from them. Often, the Do Not Attempt Resuscitation order also includes the maximum intervention agreed (13).

Limited research on Do Not Attempt Resuscitation orders has been conducted in the Gulf Cooperation Council (GCC) countries, where controversial end-of-life decisions may be compounded by ethical challenges associated with the religious and cultural beliefs of Arab patients and their families (10, 14). According to Titthecott, “*Significant steps have been taken towards coming to a consensus by Islamic scholars and under GCC State laws to ensure that patients’ wishes at the end-of-life stage of their care are taken into account to ensure their needs are met and at the same time providing legal protection for those medical practitioners at the forefront of dealing with these difficult cases*” (1).

Abbas et al. reported a significant lack of understanding about Do Not Attempt Resuscitation orders among medical students in Saudi Arabia(15). Aletreby et al. found a higher frequency of late Do Not Attempt Resuscitation orders in Saudi Arabia compared to those reported in other studies, highlighting the need for clearer guidelines to achieve consistency (16). Alalmay et al. suggested that more research is needed to provide a comprehensive understanding of the factors that predict Do Not Attempt Resuscitation decisions under Islamic law (17).

The Mobile Healthcare Service (MHS) was a community-based, public entity, providing acute and chronic healthcare to patients in their homes in the State of Qatar from 2014 to 2020. The MHS facilitated early supported discharge from hospitals for elderly, frail, and multi-comorbid patients in Qatar, through planned and unplanned home visits by family physicians, nurses, and paramedics (18). It was comprised of primary care physicians, nurses, and paramedics. Patients were visited in their homes by either nurses or physicians in the company of paramedics. Paramedics, who were from Tunisia and Jordan, would also provide Arabic-English translation when required as a significant proportion of the patients were primarily Arabic speaking. Due to the type of patient population visited by MHS clinicians, the discussion of Do Not Attempt Resuscitation orders could be regarded as an important aspect of patient care. This was made even more pertinent during the COVID-19 pandemic; however, it was not a topic that had been explored in the department. Upon detailed review of the available literature, it was ascertained that there had been minimal research conducted regarding out of hospital Do Not Attempt Resuscitation orders in the State of Qatar and the Middle East region. It was hence decided to explore the perspectives and experiences of MHS clinicians in having Do Not Attempt Resuscitation discussions in a community setting in Qatar and ascertain if the COVID-19 pandemic affected their behaviors when considering these sensitive conversations.

The aim of this descriptive cross-sectional survey was to explore the perceptions and experiences of healthcare professionals in the Mobile Healthcare Service regarding Do Not Attempt Resuscitation discussions. The objectives were to compare statistically the self-reported responses between MHS paramedics, physicians, and nurses. The survey was conducted during the COVID-19 pandemic in 2020, when elderly patients in the State of Qatar were at risk of mortality should they become infected.

## Materials and methods

This is a researcher-developed survey-based study which was approved by the HMC Medical Research Centre Institutional Review Board under expedited review (MRC-05-254) and was conducted between July 2020 and November 2020. Aiming to recruit a convenience sample involving as many MHS clinical staff as possible, all clinical staff (*N* = 106) were invited by SMS and email correspondence to participate in an online survey. The survey consisting of 18 items was developed by the research team through an iterative process to ensure clarity and validity with regards to the study objectives and was hosted on Survey Monkey. The process involved several cycles of testing the survey on multiple individuals and feedback was reviewed to confirm clarity and validity. The responses were analyzed using IBM SPSS version 22.

The frequency distributions of items with categorical responses were summarized using counts and percentages. The items with normally distributed numerical responses were summarized using mean scores ± standard deviations. The response data were analyzed statistically assuming that “*The decision-theoretic approach to hypothesis testing suggested by Neyman and Pearson is disappearing from use in major medical journals, and the practice of dividing results of hypothesis tests into “significant” and “non-significant” is outdated and unhelpful”* (19). The relationship between the variables in this study being important, their effect size needs to be considered (20). Accordingly, a clinically important association between categorical variables was indicated by Cramer’s *V* ≥ 0.25 (21) and an important difference between the smallest and largest mean scores was indicated by an effect size Cohen’s *d* ≥ 0.41 (22).

The HCPs in this study were a heterogeneous group, comprising family physicians, nurses, and paramedics. They constituted the MHS and provided home-based primary care to a largely elderly, Muslim, Arabic-ethnic patient group in Qatar. The nurses were Filipino and had all trained in the Philippines. The physicians were a multi-ethnic group, who had successfully completed their Specialist training in General Practice from either UK, Ireland, Australia or New Zealand. Lastly, the paramedics, were predominantly from Tunisia, Muslim and primarily fluent Arabic speakers, who also spoke French and English.

Within the MHS there were two types of mobile medical units; a nurse-led unit, consisting of a nurse and a paramedic and a Doctor-led unit, consisting of a physician and a paramedic. These two types of medical units operated autonomously and largely independently of each other. The nurse-led visits primarily provided wound care and intravenous (IV) antibiotics, while the doctor visits were more focused on chronic disease management and acute unplanned visits to acutely unwell patients.

It is also important to note that the MHS had piloted a system of assigned physicians to patients for approximately a quarter of its service, but that the majority of the physicians saw different patients daily and clinical continuity was an ever-present logistical challenge.

## Results

A total of 91 MHS clinicians completed the survey, and this included 38 nurses, 33 physicians, and 20 paramedics. This represented an 85.84% participation rate and is hence sufficient to provide a reliable perspective of the staff’s perception of the subject of interest.

Table 1 reveals the demographics of the staff who worked for MHS; respondents answered questions that asked the number of years each individual worked for the MHS, the age group that the respondent belonged to, and the option to state their religious beliefs. The responses are included in Table 1.

**Table 1:** Demographic profile of survey respondents.

Item	Physicians (*N* = 33)		Nurses (*N* = 38)		Paramedics (*N* = 20)	
	*n*	%	*n*	%	*n*	%
Years working for the MHS						
0 to 2	6	18.2	2	5.3	0	0.0
3 to 5	11	33.3	35	92.1	11	55.0
>5	15	45.5	1	2.6	9	45.0
Did not answer	1	3.0	0	0.0	0	0.0
Age (years)						
20 to 30	0	0.0	8	21.1	0	0.0
31 to 40	7	21.2	19	50.0	14	70.0
41 to 50	11	33.3	11	28.9	5	25.0
>50	12	36.4	0	0.0	1	5.0
Did not answer	3	9.1	0	0.0	0	0.0
Religious beliefs						
Christianity	15	45.5	34	89.5	0	0.0
Muslim	9	27.3	3	7.9	19	95.0
Hindu	2	6	0	0.0	0	0.0
Did not answer	1	3	1	2.6	1	5.0
Prefer not to say	6	18.2	0	0.0	0	0.0
Language fluently spoken						
English	33	100.0	38	100.0	18	90.00
Tagalog	0	0.0	34	89.5	0	0.0
Arabic	6	18.2	4	10.5	20	100.0
Hindi	2	6.1	1	2.6	0	0.0
French	2	6.1	1	2.6	16	80.0
Polish	2	6.1	0	0.0	0	0.0
Spanish	1	3.0	0	0.0	0	0.0
Urdu	1	3.0	1	2.6	0	0.0

Table 2 presents the frequency distributions of the items with categorical responses which were answered only by the physicians. Table 2 reveals that the majority of physicians did not have named patients assigned to their care, and the majority of physicians were unable to locate nor knew how to update the patients’ resuscitation status on the electronic medical record of the patient. The majority of physicians were aware that the opinion of two other physicians was required to confirm Do Not Resuscitate status.

**Table 2:** Responses to items with categorical responses provided by physicians.

Item	Physicians (*N* = 33)	
	*N*	%
Do you have named patients assigned to your long-term care		
No, I do not	21	63.8
Yes, I have less than 5	1	3.0
Yes, I have between 6–15	0	0
Yes, I have between 16–30	0	0
Yes, I have more than 30	33.3	
Prefer not to say	0	0
Are you aware where to find the resuscitation status for a patient on their electronic medical record?		
No	21	63.6
Yes	12	36.4
Do you know how to update a patients’ resuscitation status on their electronic medical record?		
No	28	84.8
Yes	5	15.2
To confirm DNAR status, the treating physician needs the opinion of two other doctors: (True)		
True	23	69.7
False	10	30.3

Table 3 presents the means and standard deviations of the physicians’ answers to the items concerning the factors that may be involved to initiate a Do Not Attempt Resuscitation discussion. It showed the most important factor for physicians when deciding to initiate a Do Not Attempt Resuscitation discussion was the patient’s current functional status and quality of life, along with the patient having an incurable illness with a limited life expectancy. The least important factor considered was the consideration of resources in the community and hospital settings.

**Table 3:** Measurement of items with numerical responses provided by physicians.

Item	Physicians (*N* = 23)	
	Mean	SD
Factors that may be involved when deciding to initiate a DNAR discussion (1 = most important factor; 6 = least important factor)		
Patient’s current functional status and quality of life	2.26	1.23
Patient has an incurable illness with limited life expectancy	2.29	1.38
Wishes of the patient/family	3.32	1.22
Likelihood of a poor outcome if patient is resuscitated	3.69	1.73
Knowing the patient and family well	4.24	1.50
Consideration of resources in the community and hospital settings	4.97	1.42

Table 4 compares the frequency distribution of the items with categorical responses between the physicians, nurses, and paramedics. It shows clear differences in the opinions of physicians, nurses and paramedics regrading whether Do Not Attempt Resuscitation discussions are “forbidden” or “unmentionable” topics for patients/families in Qatar. The table indicates the differences between the three HCP groups regarding experience of Do Not Attempt Resuscitation conversations in Qatar and also the personal experiences of these discussions. Furthermore Table 4 looked at how often a Do Not Attempt Resuscitation discussion was had by a HCP in the past year, the likelihood of having a Do Not Attempt Resuscitation conversation if barriers were addressed and how best to support HCPs in their training/competency of having Do Not Attempt Resuscitation conversations.

**Table 4:** Comparison of items with categorical responses by profession.

Item	Physicians (*N* = 33)		Nurses (*N* = 38)		Paramedics (*N* = 20)		Cramer’s *V*
	*n*	%	*n*	%	*N*	%	
Are DNAR discussions a ‘forbidden’ or ‘unmentionable’ topic for your patients/families here in Qatar?							
Yes, for the majority	22	66.7	7	18.4	8	40.0	42*
Yes, but only for a minority	5	15.2	13	34.2	10	50.0	
No, not at all	5	15.2	18	47.4	2	10.0	
No response provided	3	9.1	0	0.0	0	0.0	
Have you ever been involved in a conversation in Qatar with a patient/family regarding the patient’s resuscitation status?							
No	19	57.6	35	92.1	10	50.0	0.41*
Yes	14	42.4	3	7.9	10	50.0	
No response provided	0	0.0	0	0.0	0	0.0	
What options best describe your personal experiences of DNAR conversations?							
I was Happy with the discussion	5	15.2	0	0.0	4	20.0	0.43*
I was not happy with the discussion	4	12.1	0	0.0	1	5.0	
Patient/family were open to the discussion	4	12.1	3	7.9	2	10.0	
Patient/family were not open to discussion	5	15.2	0	0.0	2	10.0	
I felt prepared for the discussion	5	15.2	0	0.0	2	10.0	
I did not feel prepared for the discussion	3	9.1	0	0.0	0	0.0	
No response provided	21	63.6	35	92.1	12	60.0	
What action(s) are most likely to be taken if a patient warranted a DNAR discussion?							
Start the discussion with patient/family	12	36.4	3	7.9	6	30.0	0.43*
Discuss with an MHS Physician	13	24.2	28	73.7	14	70.0	
Discuss with an MHS Nurse/Head Nurse	0	0.0	22	57.9	2	10.0	
Discuss with the Director of Nursing	0	0.0	8	21.1	1	5.0	
Take no action	5	15.2	1	2.6	1	5.0	
No response provided	4	12.1	5	13.2	3	15.0	
How many times in the last year you thought about raising the issue of DNAR with a patient or their family?							
0	5	15.2	20	52.6	10	50.0	0.36*
1	2	6.1	5	13.2	3	15.0	
2 to 5	13	39.4	7	18.4	2	10.0	
>5	16	48.5	0	0.0	3	15.0	
No response provided	4	12.1	5	13.2	2	10.0	
Would you be more likely to have DNAR discussions if some of the barriers were addressed?							
Yes	23	69.7	28	73.7	11	55.0	0.22
Not sure	5	15.2	3	7.9	7	35.0	
No	1	3.0	2	5.3	0	0	
No response provided	4	12.1	5	13.2	2	10.0	
How many times in the last year have you had a DNAR conversation with a patient or their family?							
0	18	54.5	25	65.8	12	60.0	0.21
1	2	6.1	5	13.2	3	15.0	
2 to 5	7	21.2	3	7.9	3	15.0	
6 to 10	2	6.1	0	0.0	0	0.0	
No response provided	4	12.1	5	13.2	2	10.0	
Which of the following options would be useful to support your DNAR training/competency?							
Lectures or presentations	24	72.7	22	57.9	14	76.0	0.09
Team based training	22	66.7	19	50.0	10	50.0	
Role play or simulation-based training	15	45.5	22	57.9	10	50.0	
Suggested reading material	15	45.5	11	28.9	5	25.0	
No response provided	4	12.1	6	15.8	2	10.0	
Has the COVID-19 pandemic affected the likelihood of identifying patients with whom a DNAR discussion would be clinically appropriate?							
No	19	57.6	21	55.3	10	50.0	0.8
Yes	10	30.3	11	28.9	8	40.0	
No response provided	4	12.1	6	15.8	2	10.0	

*, clinically important difference.

Table 5 compares the numerical responses of MHS clinical staff by profession regarding the perceived potential barriers to engage in Do Not Attempt Resuscitation discussions with patients and their relatives. It shows some differences between the different HCP groups which will be addressed in the discussion section, but broadly the religious or cultural beliefs of the patient or family was the most important factor when discussing potential barriers when having Do Not Attempt Resuscitation conversations, conversely the HCP’s personal religious or cultural belief was deemed to be the least important barrier.

**Table 5:** Comparison of perceived potential barriers to having DNAR discussions by profession of the Mobile Healthcare Service clinical staff.

Item	Physicians (*N* = 33)		Nurses (*N* = 38)		Paramedics (*N* = 20)		Cohen’s *d*
	Mean	SD	Mean	SD	Mean	SD	
Ranked list of potential barriers when discussing DNAR with patients/families in Qatar (1 = most important factor; 6 = least important factor)							
The patient or families’ religious or cultural beliefs	2.36	1.42	1.87	1.22	2.67	1.89	0.51*
Not knowing the patients or families well enough	2.86	1.35	3.22	1.54	2.94	1.66	0.25
Lack of exposure to or experience of DNAR conversations in Qatar	3.08	1.44	3.90	1.04	3.07	1.22	0.73*
Language	3.10	1.78	2.50	1.48	4.12	1.99	0.47*
Personal religious or cultural beliefs	5.73	0.72	5.22	1.49	4.36	1.69	0.42*

*, clinically important difference.

## Discussion

The results of this study revealed that the majority of each of the three HCPs believed that Do Not Attempt Resuscitation discussions were a taboo subject in Qatar (Table 4). This perception is most likely due the fact that Qatar is a predominantly Muslim country, where the preservation of life is essential to the fundamentals of Islam (23).This aligns with the perceived greatest barrier across the three HCP groups being reported as the religious and cultural belief of the patient or their family (Table 5). The personal religious or cultural beliefs of the respondents were perceived to be the least important barrier across all three groups (Table 5).

This is a similar finding to a study which assessed the attitudes of physicians and nurses to DNAR orders in Palestine, which found that there was a positive attitude toward Do Not Attempt Resuscitation orders, and that their personal culture and religion did not change this (24). However, Madadin et al. noted that cultural standards and religious beliefs do play a role in physicians’ Do Not Attempt Resuscitation decision-making, although this was less important as compared to other clinical data (11).

On a practical level, our study highlighted the physicians’ limited knowledge of where to locate or how to update a patient’s resuscitation status on the Electronic Healthcare Record (EHR). There was also significant ignorance of the local organization-level policy on Do Not Attempt Resuscitation (Table 2). This lack of awareness is not unusual. Bremer et al. found that only one third of participants (Swedish physicians and nurses) had read the national ethical guidelines for CPR and that physicians had read the guidelines to a significantly lower extent than nurses (2). O’Brien et al. found that only 12% of participants in their study had read the Irish National consent policy which provides a framework for discussion of Do Not Attempt Resuscitation orders (25).

When physicians were asked to rank the factors that would most influence initiating a Do Not Attempt Resuscitation discussion, the patient’s quality of life and the presence of a terminal illness with limited life expectancy, ranked highest. Not knowing the patient or family well and consideration of resources ranked as the least important factors (Table 3). This indicates that MHS physicians were more focused on the clinical condition of the patient, rather than external factors. This finding is similar to a study in the Kingdom of Saudi Arabia by Madadin et al. that found that physicians there were more likely to decide on Do Not Attempt Resuscitation based on clinical data, such as comorbidities and previous Intensive Care Unit admissions (11).

92% of the MHS nurses had not been involved in Do Not Attempt Resuscitation discussions in Qatar (Table 4). The 8% who had been involved in a discussion said it was a positive experience, expressing the opinion that the patient/family member were open to the discussion. The low rate of nurses participating in Do Not Attempt Resuscitation discussions is very likely multifactorial. In Qatar, Do Not Attempt Resuscitation decisions are made by physicians, so the nurses may feel that they are not authorized to broach this discussion. Previous studies in other regions have concluded that nurses believe obtaining consent regarding Do Not Attempt Resuscitation orders is a physician’s responsibility (26–28). Furthermore, the MHS nurses worked independently in the community, so were unlikely to be involved in the physician-patient consultation. Interestingly, 30% of nurses had thought about raising the issue of Do Not Attempt Resuscitation with a patient/family in the preceding year and only 2% of nurses reported being unlikely not to act if they considered that a patient warranted a Do Not Attempt Resuscitation discussion (Table 4). This highlights that Do Not Attempt Resuscitation is certainly on the nurses’ radar and, similar to the findings of Raoofi et al. (29) we can say that nursing attitudes towards Do Not Attempt Resuscitation discussions are positive, since they are likely to escalate the issue to the physician if they consider a patient warrants it.

Paramedics had a significant role as medical translators and worked closely with the physicians during each home visit. This is reflected in the similar number of Do Not Attempt Resuscitation discussion experiences (Table 4) of the two groups. Paramedics had a mostly favorable response when describing their experiences of Do Not Attempt Resuscitation discussions, whereas the physicians were split between positive and negative experiences. The disparity may be due to the burden of professional and clinical responsibility felt by the physician, during the Do Not Attempt Resuscitation discussion. Pertinently, the paramedics acting as translators, were instinctively aware and attuned to the cultural nuances during these Do Not Attempt Resuscitation conversations and could translate the meaning of the message within a specific cultural context. Providing this important role as cultural broker or mediator (30) would have afforded the paramedics a comfort level and ease, not necessarily felt by all of the physicians.

Over 80% of physicians had considered raising the issue of Do Not Attempt Resuscitation with a patient’s family member in the previous year, with just 15% of physicians stating they would not take any action. 40% of nurses had considered that their patient may warrant a Do Not Attempt Resuscitation discussion and reassuringly 95% of nurses would act on this, either by discussing with a team member or initiating the conversation themselves. Half of the paramedics had thought about raising the issue of Do Not Attempt Resuscitation (Table 4). We can garner from the above responses that the staff were largely willing to consider Do Not Attempt Resuscitation conversations with patients/family members but, perhaps, overall, there was a lack of confidence in initiating the conversation themselves or other perceived barriers. This is considered by all three groups when asked if they would be more likely to have a conversation about Do Not Attempt Resuscitation if some of the barriers were addressed with a clear majority in all three groups responding in the affirmative (69.7, 73.7, and 55% for physicians, nurses, and paramedics respectively) (Table 4).

When we examined potential barriers and asked the MHS staff to rank these barriers, we found that the greatest barriers across all three groups were perceived to be the religious or cultural beliefs of the patient and/or their family, along with the factor that the staff felt they did not know the patient or their family well enough. From the physicians’ side, this could be mitigated by having named patients assigned to their long-term care and thus improving continuity of care. This study revealed that just over one third of physicians had named patients assigned to their long-term care. This lack of continuity for the majority of physicians may have posed a challenge to establishing a long-term, trusted, therapeutic relationship in which a sensitive topic as Do Not Attempt Resuscitation could be broached.

A lack of exposure or experience of Do Not Attempt Resuscitation conversations in Qatar and potential language barriers were seen as medium levels barriers when having a Do Not Attempt Resuscitation discussion with patients and/or their families (Table 5), with the primarily Tagalog- and English-fluent nursing group ranking language as a slightly more significant barrier than the other two groups. Language was perceived to be less of a barrier for physicians and paramedics than for nurses. This may be due in part to the fact that some physicians were fluent Arabic speakers (Table 1), in addition to the fact that non-Arabic speaking physicians were confident in the skills of their paramedic as a translator and cultural broker.

Across all three groups, the respondents personal religious or cultural beliefs were perceived to be the least significant barrier when deciding to discuss Do Not Attempt Resuscitation (Table 5), indicating that personal biases are not a factor when approaching this sensitive topic.

The findings of this study are consistent with previous studies reporting that many barriers still exist to hinder the process of producing a Do Not Attempt Resuscitation order (6–9). Moreover, ethical challenges are associated with the religious and cultural beliefs of Arab or Muslim patients and their families (10, 14).

The preferred methods to support or enhance Do Not Attempt Resuscitation training and competency included lectures, presentations, team-based training and role plays (Table 4). Being provided with a list of suggested reading material was much less favored, so it appears the topic is best addressed in a group learning situation allowing the opportunity for discussion, which would be in keeping with the nuanced and sensitive topic of Do Not Attempt Resuscitation conversations.

The findings and recommendations of this study are generally congruent with the systematic review of 83 worldwide studies on the production of Do Not Attempt Resuscitation orders conducted by Scholz et al. (31) which concluded that *“Clinicians need more training to address the lack of skills to overcome interactional difficulties. Attention is also needed to address issues in the organizational contexts in which such communication occurs”* (p. 1913).

Overall, for all three groups, the COVID-19 pandemic did not affect the likelihood of identifying patients with whom a Do Not Attempt Resuscitation discussion would be clinically appropriate (Table 4). This, perhaps, could be attributed to the low COVID-19 related death-rate in Qatar, particularly in the first year of the pandemic when this survey was undertaken (32). Furthermore, there were less face to face consultations by MHS during the pandemic, which like many other healthcare service providers adopted teleconsultations due to Infection Risk Control measures (33, 34).

The main recommendations garnered from this study is that HCPs would like to be provided with more training to ensure a comfort and competency when addressing Do Not Attempt Resuscitation questions with patients and their families. We would furthermore recommend that future studies should look into optimal ways to approach Do Not Attempt Resuscitation conversations with different religious and cultural groups.

The study has several strengths. Notably, the survey was issued and responded to during the height of the COVID-19 pandemic, therefore responses from participants were essentially provided in “real-time” reducing the risk of memory bias. The study also directly asked questions regarding personal experiences and attitudes of HCPs meaning it was a primary source of information regarding the HCPs experiences and attitudes, and furthermore the survey asked these same HCPs what format of training would be best suited to addressing the barriers surrounding this sensitive topic.

Weaknesses of the study that must be acknowledged include the fact the study surveyed the specific group of HCPs who worked for the Mobile Healthcare service in Qatar which was a unique service. Therefore, the responses from participants may not be replicable to other health services and we acknowledge that further studies would be useful to clarify this.

## Conclusion

This study, of experiences and perceptions of having Do Not Attempt Resuscitation discussions with patients during the COVID-19 pandemic, was nuanced by the fact that it reflected a community-based, home-visit service in Qatar, the Mobile Healthcare Service, which primarily cared for multi-comorbid, elderly, Muslim patients of Middle Eastern ethnicity. The HCPs constituting the MHS mobile units were also distinctive, with Filipino nurses, predominantly Tunisian paramedics, and multi-ethnic Western trained physicians. Bridging the cultural and religious differences between patients and HCPs in this context, is a challenge.

Our study showed a willingness and positive attitude to Do Not Attempt Resuscitation discussions, but a mindfulness of the religious and cultural beliefs of the patients with a reluctance to offend their beliefs and values. It also highlights that it helps to know the patient/family well which allows for a foundation of mutual familiarity and trust to be in place when having these types of sensitive discussions. Engaging in a Do Not Attempt Resuscitation discussion with a patient or family members can be challenging.

This study was based on the responses from three distinctive groups of clinicians working for the MHS in the community in Qatar, each having a strong association with a different nationality and culture, often different from that of the patients they cared for.

Finally, in alignment with several cited studies, it highlights the importance of organization-level training around Do Not Attempt Resuscitation.

## Data availability statement

The original contributions presented in the study are included in the article/supplementary material, further inquiries can be directed to the corresponding author.

## Ethics statement

The studies involving human participants were reviewed and approved by the Medical Research Committee, Hamad Medical Corporation (MRC-01-20-433). Documentation of informed consent was waived by the HMC-IRB as per institutional and local requirements.

## Author contributions

AF devised the research, assisted by CF. Along with LA, and with guidance from GA throughout they formatted the methods and materials. AF, LA, and CF were all involved in collecting the data, and the original draft and subsequent edits were written by AF with assistance from CF, LA, and GA throughout. AH assisted with the final draft in particular the management of the references.
