# Corrigendum: Perceptions and experiences of community-based healthcare professionals in the state of Qatar having do not attempt resuscitation discussions during the COVID-19 pandemic

**DOI:** 10.3389/fmed.2024.1376376

**Published:** 2024-02-16

**Authors:** Audrey Fitzgerald, Conor Fitzgerald, Louise Anderson, Ammar Ali Hussain, Guillaume Alinier

**Affiliations:** ^1^Hamad Medical Corporation, Home Healthcare Services, Doha, Qatar; ^2^Hamad Medical Corporation Ambulance Service, Doha, Qatar; ^3^University of Hertfordshire, Hatfield, United Kingdom; ^4^Weill Cornell Medicine-Qatar, Doha, Qatar; ^5^Northumbria University, Newcastle upon Tyne, United Kingdom

**Keywords:** do not attempt resuscitation, COVID-19 pandemic, community medicine, Qatar, religion

In the published article, there was an error in the Ethics statement. The correct Ethics statement appears below.

## Ethics statement

The studies involving human participants were reviewed and approved by the Medical Research Committee, Hamad Medical Corporation (MRC-01-20-433). Documentation of informed consent was waived by the HMC-IRB as per institutional and local requirements.

The authors apologize for this error and state that this does not change the scientific conclusions of the article in any way. The original article has been updated.

